# Supplementary material for: Pharmacological Evaluation of Araliadiol as a Novel Anti-Inflammatory Agent in LPS-Induced RAW 264.7 Cells
Source: Biomedicines. 2025 Jun 8;13(6):1408. doi: 10.3390/biomedicines13061408 (PMC12190465; doi:10.3390/biomedicines13061408)

Figure S1. Evaluation of LPS-induced cytotoxicity in RAW 264.7 cells. **(a)** RAW 264.7 cells were seeded into 96-well plates (1×10^4 cells/well) and incubated for 24 h. Cells were then treated with LPS (0–1,000 ng/mL) for up to 48 h. Cell viability was determined using an ATP-content assay. **(b)** Cells were seeded into 12-well plates (1×10^5 cells/well) and incubated for 24 h. Following treatment with the LPS (0-1,000 ng/mL) for up to 48 h, cytotoxicity was evaluated using a crystal violet staining assay. Data are presented as mean ± SD from three independent experiments. Statistical analysis was performed using one-way ANOVA followed by Tukey's post hoc test. $^{\#}p < 0.05$; $^{\#\#\#,***}p < 0.001$ compared with the solvent-treated vehicular control group.

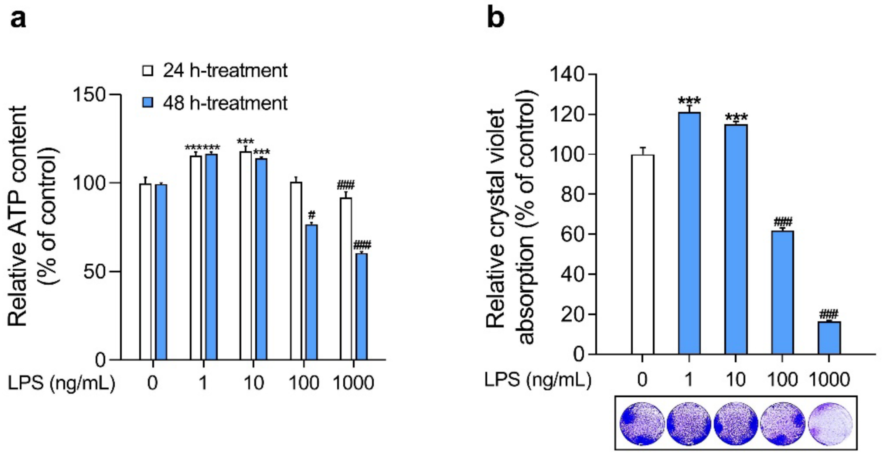

Supplement: Supplementary file 1 [file biomedicines-13-01408-s001.zip › biomedicines-3641755-supplementary.pdf]
